# Optimizing of the Cementitious Composite Matrix by Addition of Steel Wool Fibers (Chopped) Based on Physical and Mechanical Analysis

**DOI:** 10.3390/ma14051094

**Published:** 2021-02-26

**Authors:** Akrm A Rmdan Amer, Mohd Mustafa Al Bakri Abdullah, Yun Ming Liew, Ikmal Hakem A Aziz, Jerzy J. Wysłocki, Muhammad Faheem Mohd Tahir, Wojciech Sochacki, Sebastian Garus, Joanna Gondro, Hetham A. R. Amer

**Affiliations:** 1 Geopolymer & Green Technology, Centre of Excellence (CEGeoGTech), Universiti Malaysia Perlis (UniMAP), Perlis 01000, Malaysia; ymliew@unimap.edu.my (Y.M.L.); ikmalhakem@unimap.edu.my (I.H.A.A.); faheem@unimap.edu.my (M.F.M.T.); 2 Civil Engineering Department, Omar Al Mukhtar Universiti, Al Baida 991, Libya; haithm_82@yahoo.com; 3 Faculty of Chemical Engineering Technology, Universiti Malaysia Perlis (UniMAP), Perlis 01000, Malaysia; 4 Department of Physics, Częstochowa University of Technology, 42-201 Częstochowa, Poland; wyslocki.jerzy@wip.pcz.pl (J.J.W.); joanna.gondro@pcz.pl (J.G.); 5 Faculty of Mechanical Engineering and Computer Science, Częstochowa University of Technology, 42-201 Częstochowa, Poland; w.sochacki@imipkm.pcz.pl (W.S.); gari.sg@gmail.com (S.G.)

**Keywords:** steel wool fiber, chopped fiber, cement composite

## Abstract

The demand for durable, resistant, and high-strength structural material has led to the use of fibers as reinforcing elements. This paper presents an investigation into the inclusion of chopped steel wool fibers (CSWFs) in cement to form a high-flexural strength cementitious composite matrix (CCM). CSWFs were used as the primary reinforcement in CCM at increments of 0.5 wt%, from 0.5–6 wt%, with ratios of cement to sand of 1:1.5 and water to cement of 0.45. The inclusion of CSWFs resulted in an excellent optimization of the physicomechanical properties of the CCM, such as its density (2.302 g/cm^3^), compressive strength (61.452 MPa), and maximum flexural strength (10.64 MPa), all of which exceeded the performances of other reinforcement elements reported in the literature.

## 1. Introduction

The construction industry has begun increasing its use of composites as structural or reinforcement materials. Cementitious composites are typically brittle and have low tensile strength and strain capacities [1,2,3,4], quickly developing cracks from high applied loads, thus limiting applicability in handling high service loads and resulting in collapsed structures due to the lack of post-peak resistance.

The weakness of concrete can be overcome through the addition of reinforcing bars or prestressed steel. However, the latter’s production negatively impacts the environment via iron bar mining, which is energy intensive and pollutes the air due to the release of nitrous oxide, carbon dioxide, carbon monoxide, and sulfur dioxide from diesel generators, trucks, and other equipment. Therefore, alternatives are highly sought after to minimize damage to the environment [5,6]. Fiber-reinforced concrete/cement (FRC) is concrete or cementitious material reinforced with short and dispersed fibers of lengths ~1–75 mm. ACI544 defines FRC as concrete consisting of hydraulic cement, aggregates, and discrete reinforcing fibers. Fibers suitable for reinforcing concrete have been produced from steel, glass, and organic polymers [7]. Naturally-occurring asbestos fibers and vegetable fibers, such as sisal and jute, can also be used for reinforcement. The matrix can be concrete, mortar, or cement paste. 

The production of chopped steel wool fibers (CSWFs) through recycling can alleviate the aforementioned environmental issues and promote green technology in the construction industry [8,9]. The future of the industry involves recycling, and CSWFs, commonly made of low-carbon steel, can be used in the form of rolls or pads in brake pad linings (as a thermal shield); in automobile exhaust pipes (as an acoustic shield); in metal separation, water, and air filtration; and in cleaning. The cost of CSWFs is ~30–50% of the cost of steel fibers and they can be produced from recycled metal scraps [10]. The presence of steel fibers in high-strength cementitious composites and conventional concrete prevents crack formation. However, the influence of CSWFs on the physicomechanical properties of a cementitious composite matrix (CCM) has only seen limited investigation in the last decade [11].

Aldikheeli et al. [12] investigated the effect of the inclusion of hooked-end steel fibers on the density of cement mortar using two diameters and lengths. It was reported that the dry densities of the composites exceeded that of their conventional counterpart, increasing to ~5.4% at a concentration of steel fibers of 1.5%. The results also showed that the water absorption of the conventional samples exceeded that of the reinforced mortar.

Zinkaah [13] found that the water absorption of lightweight concrete with steel fibers exceeded its counterpart lacking fibers, increasing from 1.31% to 9.24% when the volume of steel fibers increased from 0 to 1%. Moreover, Jhatial et al. [14] elucidated the effect of hooked-end steel fibers with lengths of 25 mm and diameters of 0.5 mm on the compressive and flexural strengths of the composite and reported that a 3% steel fiber fraction reinforcement resulted in the highest compressive strength of 31.46 MPa, which was ~18.27% higher than that of the control sample; its flexural strength was 6.16 MPa, which was ~51.72% higher than that of the control sample.

Many studies have been published in the literature discussing the addition of steel fibers in ordinary Portland cement (OPC) concrete, which resulted in increased flexural and compressive strengths [15,16,17,18], attributed to the steel fibers’ reinforcement. Table 1 summarizes previous studies involving steel fibers as the primary reinforcement in concrete composites and their effect on the flexural and compressive strengths.

Despite the prevalence of studies in the literature discussing steel reinforcement in composites, there are many open research questions on the subject, such as those concerning the correlations between the cementitious matrices and CSWF inclusion and the optimal additions of CSWFs to obtain the best cement-based composites. The paper details an investigation of CSWF concentrations in a cementitious composite matrix.

## 2. Materials and Methods

### 2.1. Materials

The matrix was produced using OPC as its binder. Cement (CIMA, Perlis, Malaysia), water, and fine sand (LSK Enterprise Sdn. Bhd., Perak, Malaysia), were combined to produce the mortar. CSWFs were used as the primary reinforcement material. The OPC was manufactured by Cement Industries of Malaysia Berhad (CIMA), Perlis, Malaysia and is called Lion Blue ASTM Type I Portland Cement. It is the most common type of cement and is used in general concrete construction. Lion Blue OPC is manufactured under an effective testing system involving control, monitoring, and compliance with the MS EN 197-1:2007. Its physicochemical properties are detailed in Table 2.

**Figure 1:**
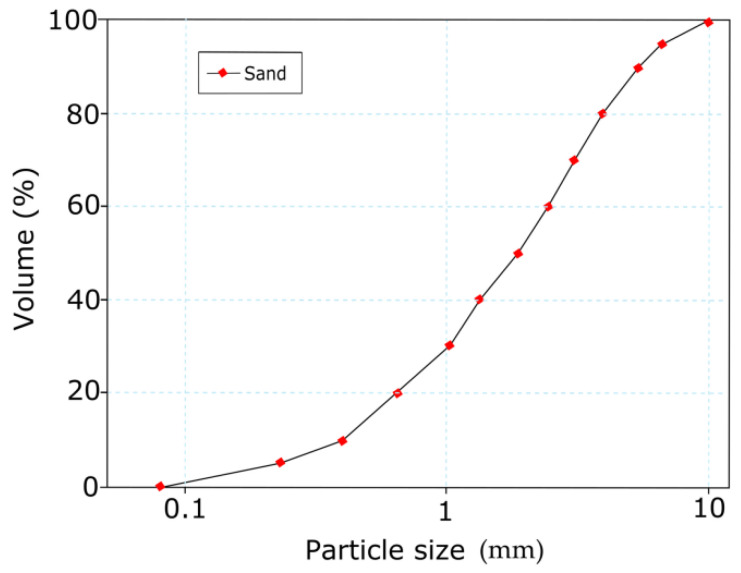
Particle size distribution of sand.

The sand was used as a filler to produce matrix mortar in this study. The fine aggregate used was quartz river sand obtained from a local supplier in Perlis. The proper distribution of sand particles significantly impacts the physicomechanical properties of a matrix. The detailed particle size distributions of sand are shown in Figure 1 [19]. Fine aggregates with diameters of 9.5 mm ≤ sand size < 75 µm were used to improve the slurry’s filling performance and minimize material separation. Coarse aggregates were avoided to ensure that harmful factors such as coal, lignite, organic impurities, silt, and powdered clay were absent from the matrix. The fine aggregate was washed with clean tap water, placed on a plastic sheet, and dried at room temperature. The dry-condition fine aggregate was used in this research to produce the matrix.

The CSWFs were produced from recycled scrap metal obtained from Rahamatullah Sdn Bhd (Kedah, Malaysia) and used as reinforcement materials. The fibers’ characteristics are detailed in Table 3. The correlation coefficient was used to represent the correlations between the CSWFs inclusion quality as a variable parameter with the density, water absorption, compressive strength, and flexural strength of the generated cementitious composite.

### 2.2. Preparation of Cementitious Composite Matrix

The CSWF-reinforced cementitious composite matrix was prepared by mixing the steel fibers as the reinforcement into the composite at a ratio of 0.5–6.0 and at an increment of 0.5, with constant ratios of cement to sand of 1:1.5 and water to cement of 0.45.

A total of 12 CSWF-reinforced cement mortar mix designs were produced as control samples. For every design, three samples were produced and the results from the tests were averaged to minimize errors. The fresh mixture was poured from the mixer into the pre-oiled mold after mixing. There were two molds used in this research: a cube of 50 mm × 50 mm × 50 mm uses for density, water absorption, and compressive strength testing; and a beam mold used for flexural strength, with dimensions of 25 mm × 100 mm × 500 mm. A total of 72 samples were prepared and tested after curing for 28 days.

### 2.3. Testing and Characterization of Cementitious Composite Matrix

#### 2.3.1. Density and Water Absorption Measurement

The CSWF cement composite specimens were poured and compacted using a vibrating table at a frequency of 50 Hz. The molds were half-filled and vibrated for 10 s, and then fully filled and vibrated for 15 s more [20], in a moist room at 23 ± 2 °C until demoulding 24 h later [21]. After this, the specimens were kept in a controlled temperature water tank (21 ± 2 °C) for 24 h until testing. The samples were removed from the water and allowed to drain for a minute; any visible water was removed with a damp cloth. After that, the samples were weighed and their saturated weight (W_s_) was recorded. Then, the samples were dried in an oven at a temperature of 110 °C for 24 h. After that, the dried samples were weighed and the dried oven weight (W_d_) recorded [22]. Following ASTM C140, the density and water absorption were calculated using Equations (1) and (2):

(1) $$\mathrm{Density}\;=\;\frac{W_{d}}{\mathrm{Volume},\;V}$$

and

(2) $$\mathrm{Water}\;\mathrm{absorption}\;\left(\%\right)=\;\frac{W_{s}-W_{d}}{W_{d}}\times 100$$

#### 2.3.2. Compressive Strength Test

The compressive strength was evaluated using a SHIMADZU AG-Xplus machine (AG-20/50kNX/50kNXDplus, Duisburg, Germany) with a loading capacity of 250 KN, using a speed of 5 mm/min. Compressive strength was performed on cubes with dimensions of 50 mm × 50 mm × 50 mm, following ASTM C109. The maximum load was recorded and the strength was calculated by dividing the maximum load by the area of the face subjected to loading. The strength was recorded in N/mm^2^ or MPa. The compressive strength was calculated using the formula shown in Equation (3):

(3) $$\mathrm{Compressive}\;\mathrm{strength}=\frac{P\left(\mathrm{Load}\right)}{A\left(\mathrm{Area}\right)}$$

#### 2.3.3. Flexural Strength Test

**Figure 2:**
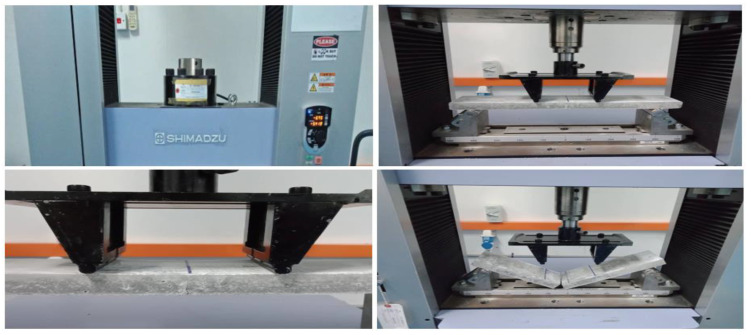
Third point loading procedure.

The flexural ultimate strength for a thin-section beam was assessed with a third point loading (TPL) test [12,13,14,15,16]. The constant displacement rate used in this experiment was 2 mm × min^−1^. The diameters of the upper support and lower support were not less than 12.7 mm, using a simple beam of 25 mm in depth such that the test specimen had a ratio of the specimen major span (the length span between the lower supports) to the specimen depth of 16 to 1. The setup procedure is shown in Figure 2.

The ultimate flexural strength was calculated by measuring the specimen depth and width after flexure for each of the non-reinforced and the reinforced samples; the calculation formula was as shown in Equation (4):

(4) $$F_{u}=\frac{P_{u}\;L}{bd^{2}}$$

where *F_u_* is the flexural ultimate strength (MPa); *P_u_* is the ultimate load (N), which is the maximum force achieved by the specimen; *L* is the major support span (mm); *b* is the width of the specimen (mm); and *d* is the depth of the specimen (mm).

#### 2.3.4. Least Squares Regression

The correlation coefficient is a statistical measurement of the strength of the relationship between the relative movements of two variables. A correlation coefficient with an absolute value of R^2^ ≥0.9 would represent a very strong relationship. Using least squares regression (LSR), the linear equations and the correlation coefficient were assessed by the following equations:

$$y=a_{0}+a_{1^{x}}$$

where:

*a*_1_ is the slope;
*a*_0_ is the intercept.

$$a_{1}=\frac{n\sum x_{i}y_{i}-\sum x_{i}\sum y_{i}}{n\sum x_{i}^{2}-{\left(\sum x_{i}\right)}^{2}}$$

$$a_{0}=\bar{y}-a_{1}\bar{x}\;\mathrm{or}\;a_{0}=\frac{\sum y_{i}-a_{1}\sum x_{i}}{n}$$

where:

*n* is the number of experiment values;
$\bar{y}$ is the mean of $y_{i}$; and
$\bar{x}$ is the mean of $x_{i}$.

Furthermore, the LSR procedure can be readily extended to fit the data to a higher-order polynomial, as follows:

$$y=a_{0}+a_{1}x+a_{2}x^{2}+\cdots +a_{m}x^{m}$$

The correlation coefficient (*R*) can be calculated by:

$$R=\frac{n\sum x_{i}y_{i}-\left(\sum x_{i}\right)\left(\sum y_{i}\right)}{\sqrt{[n\sum x_{i}^{2}-\left(\sum x_{i}{)}^{2}\right][n\sum y_{i}^{2}-\left(\sum y_{i}{)}^{2}\right]}}$$

#### 2.3.5. Optical Microscope

The morphology study was conducted using an optical microscope (OM) from Olympus Optical Co. Ltd., model BX41RF, Tokyo, Japan. Microstructure analysis of the cross-section was performed to investigate the void distribution and sizes, the fiber distribution, and the orientation and levels of the agglomeration spots.

## 3. Results and Discussions

### 3.1. Physical Analysis of Reinforced Cementitious Composite Matrix

#### 3.1.1. Density

**Figure 3:**
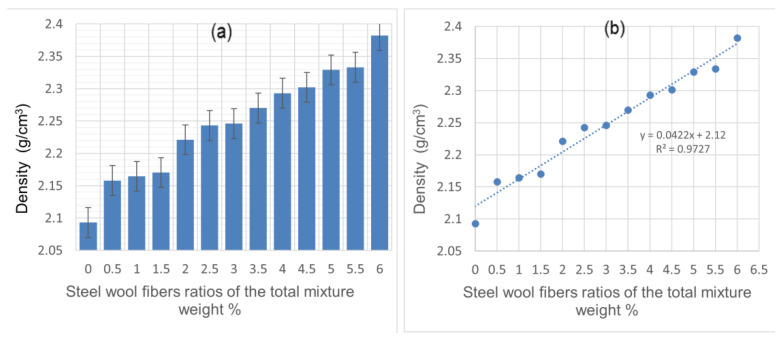
The density of cement mortar for various ratios of CSWFs: (**a**) density values of the composite matrix corresponding to the CSWF ratios, (**b**) correlation between density and CSWF addition.

Figure 3a shows the cement mortar density for various concentrations of CSWFs (0–6.0%). Increased concentrations of CSWFs resulted in increased overall composite densities. It can be seen that the density was 2.093 g/cm^3^ at 0%, 2.243 g/cm^3^ at 2.5%, 2.302 g/cm^3^ at 4.5%, and 2.382 g/cm^3^ at 6.0%, with the density increasing at a rate of 7.17%, 9.99%, and 13.81%, respectively, due to the inclusion of CSWFs.

Figure 3b shows the linear relationship between the concentration (and presence) of CSWFs and the mortar’s density, resulting in the linear equation y = 0.0422x + 2.12, where y is the density (g/cm^3^) and x is the ratio of CSWFs to the total mixture weight. An increase in the fiber fraction of 0.1 can be expected to increase the density by 0.2% (g/cm^3^), as per the linear equation. The concrete samples’ overall densities were close to that of their conventional counterpart, within 2.2–2.6 g/cm^3^ [23]. The density increase due to the addition of CSWFs can be attributed to the latter’s specific gravity (7.8 g/cm^3^), which is much higher than standard concrete (2.4 g/cm^3^). Harshavardhan et al. [24] also reported a similar observation, with the addition of aggregates or reinforcement fibers with higher specific gravity increasing the overall density of the concrete’s composite.

#### 3.1.2. Water Absorption

**Figure 4:**
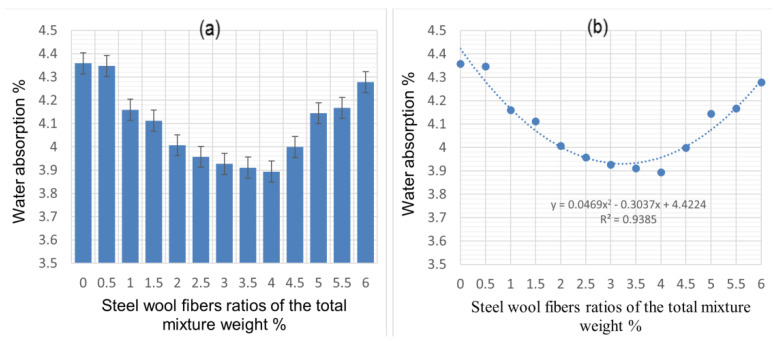
The water absorption of cement mortar for various ratios of CSWFs: (**a**) water absorption values corresponding to the CSWF ratios, (**b**) correlation between water absorption and CSWF addition.

Figure 4a shows the cement mortar’s water absorption at various CSWF ratios. It can be seen that increasing the CSWF concentration from 0.5% to 4% decreased the water absorption of the composite relative to that of the conventional mortar. Water absorption slightly increased when the concentration of CSWFs increased from 4.0% to 6.0%. The results suggest that the samples’ water absorptions were lower than that of their conventional counterpart (unreinforced cement mortar).

The water absorption of the cementitious composite matrix resulting from the addition of CSWFs at 28 days was in line with ASTM C140, according to which the average water absorption of test samples should not be greater than 5%, with no individual unit more significant than 7.0%. The lower water absorption confirms a higher resistance to water penetration and lower overall environmental damage [25], which in turn confirms the samples’ near impermeability. Abdullah et al. have reported that concrete with a porosity exceeding 5% should be classified as highly permeable [26], and low permeability indicates less porous concrete. Generally, permeability is a measure of how easy it is for water, air, and other substances, such as chloride and sulfates, to be absorbed by the concrete, making it a significant property vis-à-vis the durability of the structure [27]. The absorption is made possible via pores, and higher porosity almost always results in lower concrete density and increased water absorption, and vice versa. Although the increasing density was expected to decrease water absorption, this was not observed in the samples analyzed in this study. As per Figure 4b, the addition of fibers at concentrations lower than 4.5% decreased water absorption. When the fiber addition increased above 4%, a slight increase in water absorption was evident. This observation can be attributed to the fact that a composite’s water absorption is more closely tied to the pore structure than density.

**Figure 5:**
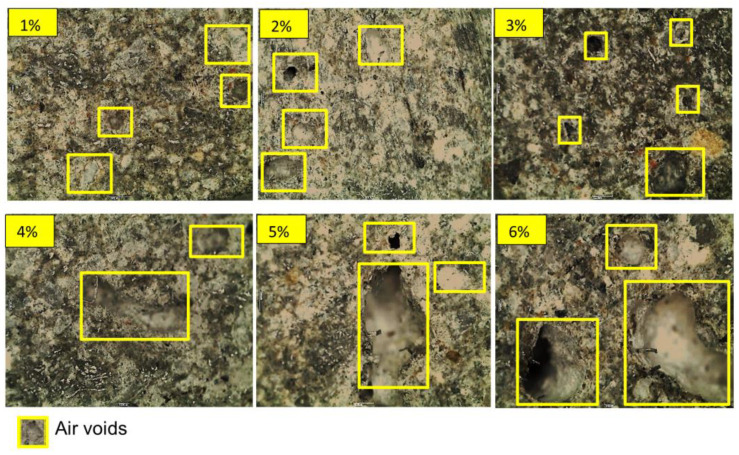
The appearance of air voids in samples with various CSWF distributions (1%, 2%, 3%, 4%, 5%, and 6%).

Figure 5 confirms that increasing the fiber fraction beyond 4% resulted in altered air void distribution and sizes in the CCM. García et al. posited that the air void content in a mixture depends mainly on the percentage of the clusters of fibers in the mixture [28], while Schutter et al. [29] noted that a more porous generated CCM results in increased degradation of a sample, caused by increased overall water absorption. Shawnim et al. [30] pointed out that the uneven distribution of large-sized pores can lead to high permeability, resulting in increased water absorption, while Ramamurthy et al. [31] observed that smaller air voids result in lower porosity and vice versa.

### 3.2. Mechanical Performance Analysis of Reinforced Cementitious Composite Matrix

#### 3.2.1. Compressive Strength

**Figure 6:**
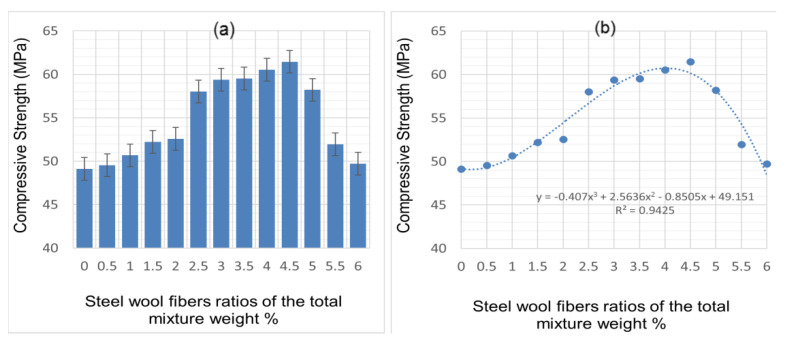
Compressive strength of cement mortar at various CSWF ratios: (**a**) compressive strength values corresponding to the CSWF ratios, (**b**) correlation between compressive strength and CSWF addition.

The effects of the inclusion of CSWFs on the cement mortar’s compressive strength are summarized in Figure 6a,b. It can be seen that the highest compressive strength resulted from the addition of 4.5% CSWFs, producing a compressive strength of 61.452 MPa. Concrete without steel wool fibers was found to result in a compressive strength of 49.124 MPa. It can also be surmised from this graph that the optimal loading, resulting in the highest compressive strength, would be 4.5%, as, beyond this concentration, the compressive strength of the cement mortar decreased.

An equation was derived using the experimental results and the correlation between the compressive strength and CSWF concentrations, relying on the plot seen in Figure 6b. A 3D polynomial equation was derived to represent the nonlinear relationship between compressive strength and concentrations of CSWFs, and this equation can be used to predict the compressive strength of hypothetical concentrations of CSWFs when an experiment cannot be conducted. 

The increased compressive strength can be attributed to the inclusion of CSWFs. Cuenca and Serna [32] reported that steel fibers bridge crack spots in OPC concrete at sufficient concentrations.

**Figure 7:**
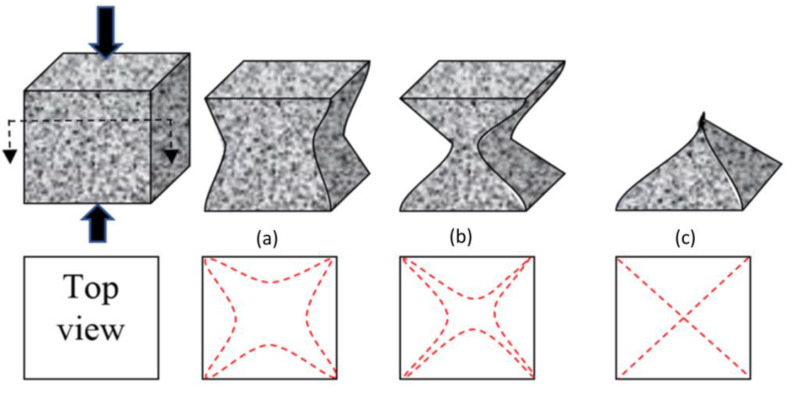
Typical crack patterns in the cube samples. (**a**) A non-explosive pattern, (**b**) a semi-explosive pattern, and (**c**) an explosive pattern.

**Figure 8:**
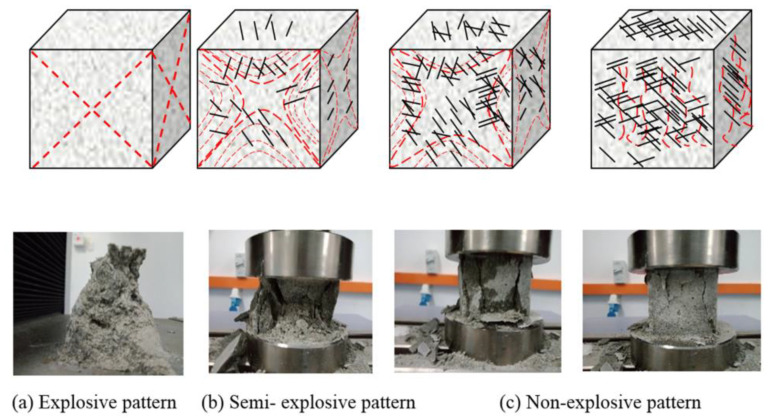
The efficiency of CSWFs with regard to the ability to bridge crack patterns. (**a**) Explosive pattern; (**b**) Semi-explosive pattern; (**c**) Non-explosive pattern.

The efficiency of fiber inclusion vis-à-vis crack mitigation can be represented with a schematic diagram (Figure 7). The application of compressive load resulted in the continuous appearance of cracks on the cubes. The typical crack patterns of the CCM were in line with those described in BS EN 12390-3 and are shown in Figure 7. Figure 8a shows images of the CCM without reinforcement exhibiting an explosive pattern at peak load due to its high brittleness. Samples that included steel fibers demonstrated a more ductile behavior and lacked the explosive patterns seen in Figure 8b,c due to the bridging effect created by the steel fibers, which distributed stresses from the compression load throughout the entire sample.

The compressive strength was reduced when fiber additions exceeded 4.5% due to the low workability of the concrete. The mixture’s air void content depends mainly on the percentage of fiber clusters in the mixture and is related to strength. According to Mehta et al. [33], pores in the composite and strength in the hardened CCM are inversely correlated, with increased porosity resulting in lower strength. Composites with a narrower void distribution are more robust relative to those with wider voids. Evidence of these internal physical alterations can be seen in Figure 4, which shows that fiber additions greater than 4.5% resulted in a larger pore size and decreased strength. Fiber additions greater than 5.0% resulted in decreased compressive strength and increasing density, an observation that can be attributed to the differences between the reinforcement density and matrix density.

#### 3.2.2. Flexural Strength

**Figure 9:**
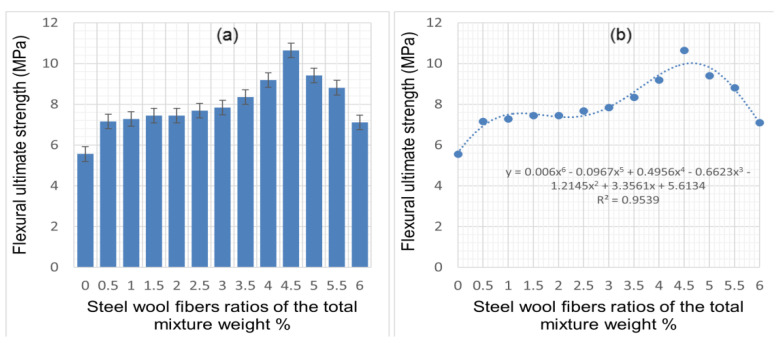
The flexural strength of cement mortar at various CSWF ratios: (**a**) flexural strength values corresponding to the CSWF ratios, (**b**) correlation between flexural strength and CSWF addition.

The capacity of CSWF-reinforced cement mortar to resist bending was quantified using a TPL test. The cement mortar specimens’ flexural strengths are detailed in Figure 9a,b.

The results show that the flexural strength of concrete improved with CSWFs, as also found for other fibers in other studies [34,35,36]. It can be seen that the optimal increase of the flexural strength was a 91.37% improvement relative to the flexural strength of mortar due to the addition of 4.5% fiber. However, it should also be pointed out that the addition of CSWFs at concentrations greater than 4.5% resulted in decreased flexural strength. The fibers enhanced the structure’s cracking response and improved its deflection capacity before failure, translating into improved overall ductility, as evidenced in Figure 9.

**Figure 10:**
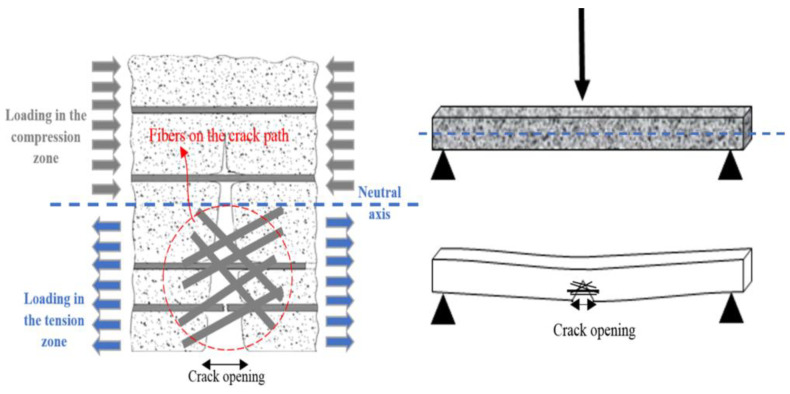
The efficiency of steel fibers with regard to the ability to bridge crack openings.

Furthermore, increasing fiber content resulted in the bridging of cracks up to a reliable limit of the load. This led to improved flexural strength with increasing CSWFs in the generated CCM relative to its conventional counterpart. Increasing fiber content resulted in increased fiber distribution across the CCM, placing it on the crack propagation paths, as shown in Figure 10. The influence of fibers on the flexural strength of concrete is mainly dependent on fiber content. The maximum increment was ~91.37% with a 4.5% addition of CSWFs. The strength reductions observed in samples with 5%, 5.55%, and 6% CSWFs were 69.25%, 58.45%, and 27.88%, respectively. This was due to the poor distribution of fibers within the concrete, causing fiber agglomeration in the samples.

Equations were proposed to predict the flexural capacities of a wide variety of beams, aiming to accurately predict the flexural strength with different addition ratios of CSWFs. These are presented in Figure 9b, where Y is the apparent flexural capacities strength (MPa) and X is the CSWF ratio of the total mixture weight. The nonlinear analysis demonstrated that the contribution to the concrete flexural test’s bending moment depends considerably on the fiber content. This is supported by Yoo et al. [20], who proved that steel fibers could bridge crack spots if the total amount of fibers was sufficient.

**Figure 11:**
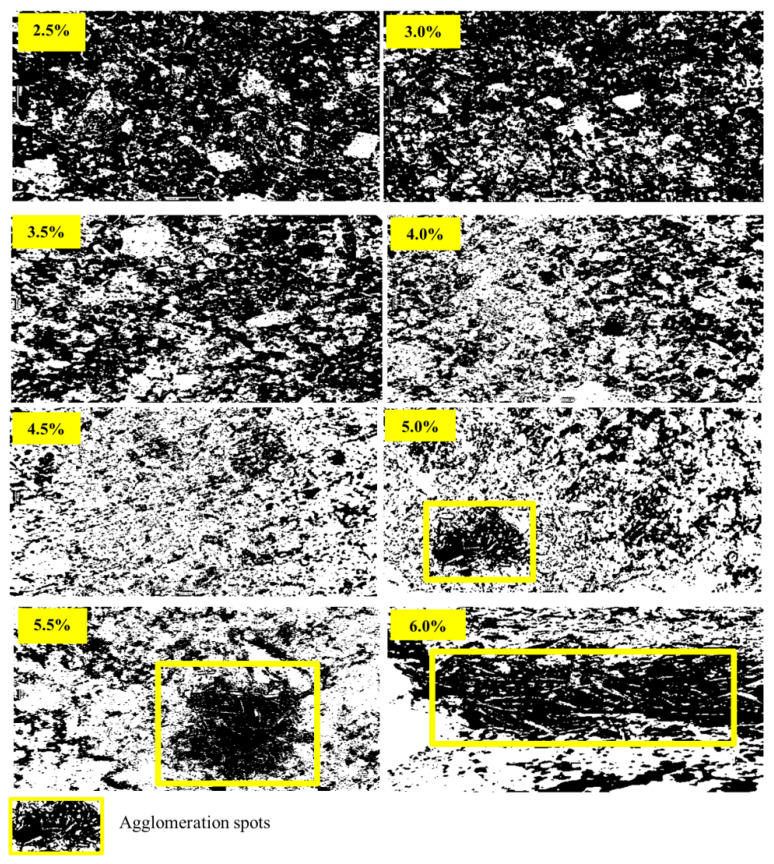
Beam cross-section images of CSWF distributions with ratios of 2.5% to 6%, cut horizontally.

Several beam specimens containing fibers were cut horizontally and vertically for microscopic analysis of their cross-sections after the TPL test. The analyses were intended to image the distribution of fibers in the composite mortar, which significantly affected the sample strength and deformability under loading. Figure 11 shows cross-section images of the horizontally cut beam samples, representing the CSWF distribution in the mortar influenced by the fiber addition. The fiber additions of 5%, 5.5%, and 6% resulted in a uniform fiber distribution. However, due to the poor workability of the fresh composites, the CSWFs faced flow difficulties, where inclusions of fibers at concentrations higher than 4.5% made it difficult for the fibers to move and disperse, creating localization and spot agglomeration, as seen in Figure 11. It can be concluded that poor workability contributed to the agglomeration of CSWFs at inclusions above 4.5%.

The bright regions in the images in Figure 11 represent the CSWFs, while the dark regions represent the samples’ binder regions. Fiber distribution affected the physical properties of the samples, especially the water absorption of the CCM. The increase of the fiber fraction beyond 4.5% increases and changes the mixture’s air void distribution due to the fibers’ agglomeration, which increases water absorption at the cost of mechanical properties, such as strength.

**Figure 12:**
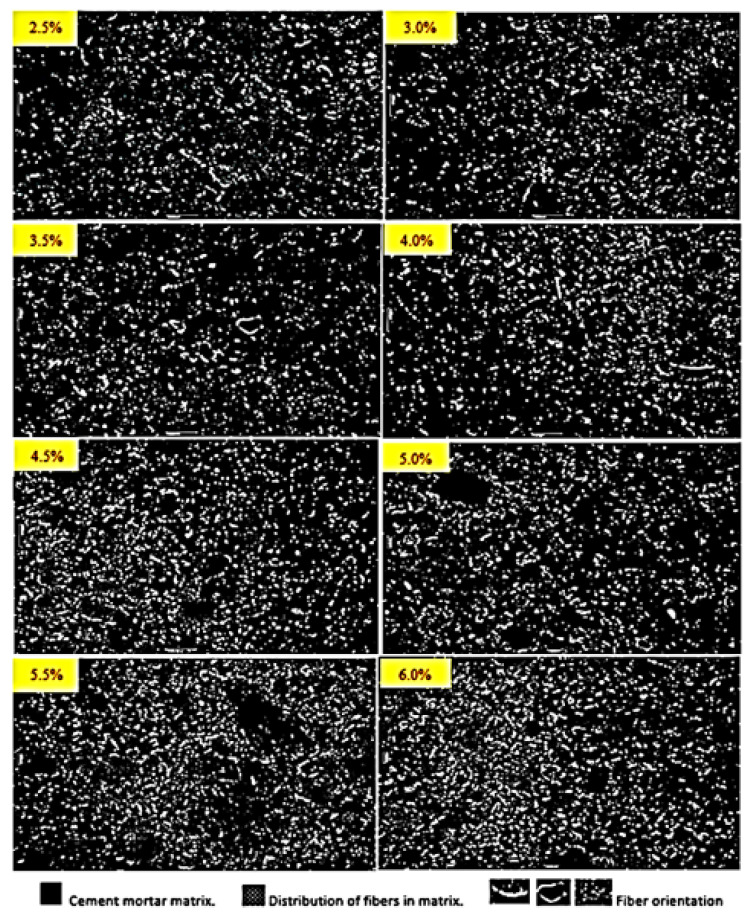
Beam cross-section images of CSWF distributions with ratios of 2.5% to 6%, cut vertically.

Furthermore, the performance of CSWFs depends on their orientation in the CCM, with the optimum mechanical properties being in the direction of reinforcement. In other words, the optimum orientation is in the same direction as the load direction, which in each case depends on different load behaviors. Regarding the load behavior, one example in the case of the beam is the tensile and compression force, which is almost perpendicular to the applied load direction of the specimens that need reinforcement along the top and the bottom. In compression, similar to the tensile response, the incorporation of steel fibers primarily augments the flexural properties. The fibers can act most effectively if aligned in the direction of the stress. Li et al. [37] pointed out that, due to the high specific gravity of steel fiber in comparison with the CCM, the fiber tends to be more horizontally than vertically orientated. Moreover, Gettu et al. [21] investigated the effect of the type of compaction, such as table vibration, hand tamping, and internal vibration, as well as the vibration time, on the steel fiber orientation. It was reported that the table vibration at a frequency of 50 Hz for molds half-filled by 10 s, and then fully filled after a further 15 s, would lead to a preferential horizontal orientation of the fibers in the same load direction. To confirm this possibility, beam specimens containing fibers were cut vertically for microscopic analysis of their cross-sections. Figure 12 shows that many fibers were distributed almost perpendicularly to the applied load direction and seem to appear nearly as geometrical points, while the rest of the fibers are notably crimped or curved in their shapes.

Furthermore, the chopped steel fibers improved pre-peak mechanical performance and strength by delaying the formation of through-specimen macro-cracks. Micro-cracks are the weakest parts in concrete. Under the influence of external forces, the micro-cracks undergo a development process; the destruction of the concrete is certainly caused by the continuous generation, expansion, and destabilization of the micro-cracks. The closely spaced fibers interact with cracks at the microstructural level and hamper the widening of coalesced micro-cracks, thus encouraging the growth of multiple cracks. This behavior can improve the initial flexural modulus and the energy absorption, as well as providing some improvement in ductility. As a result, the incorporation of steel fibers can delay the formation of micro-cracks through the specimens. However, steel fibers cannot change the generation of the macro-cracks due to the short fiber lengths.

## 4. Conclusions

The optimizing of the mechanical performance of a cementitious composite matrix through the addition of chopped steel wool fibers was experimentally investigated in this paper. The effects of chopped steel wool fibers on density, water absorption, compressive strength, and flexural strength were examined. The samples with steel fibers exhibited better characteristics relative to those lacking fibers. It was also confirmed that the optimal loading of CSWFs was at a concentration of 4.5%, which resulted in the optimum compressive strength and flexural strength. The maximum increase rates were 25.1% for the compressive strength and 91.37% for the flexural strength. The objective of this research was achieved through the discovery of the optimal loading of steel fibers in the composite, where the inclusion of a concentration of 4.5 wt% CSWFs in a CCM increased its density to 2.302 g/cm^3^, optimized its compressive strength at 61.452 MPa, and maximized its flexural strength to 10.64 MPa.

## Figures and Tables

**Table 1:** Summary of relevant studies featuring steel fibers as the primary reinforcement in concrete composite based on the flexural and compressive strength.

Author	Types of Concrete	Steel Fibers Type	SF%	Flexural Strength (MPa) 28 days		Compressive Strength (MPa) 28 days	
				with SF	without	with SF	without
Aldikheeli & Shubber, 2020 [12]	Cement-based	hooked-end length of 35 mm, diameter of 0.55 mm	1.50	4.40	4.20	31.46	26.00
		hooked-end length of 60 mm, diameter of 0.90 mm	1.50	6.90	4.20	30.00	26.00
Zinkaah, 2014 [13]	Cement-based	Straight length of 15 mm, diameter of 0.2 mm	1.00	10.18	6.60	38.80	29.77
Jhatial et al., 2018 [14]	Normal concrete (M20), OPC	Hooked-end length of 25 mm, diameter of 0.5 mm	3.00	6.16	4.09	31.46	26.60
Iqbal et al., 2015 [15]	Cement-based	Straight length of 13 mm, diameter of 0.2 mm	1.25	7.62	-	59.74	-
Mahadik et al., 2014 [16]	Normal concrete (M40), OPC	Straight length of 60 mm, diameter of 0.75 mm	0.75	6.62	4.62	51.45	41.42

**Table 2:** Physicochemical properties of the ordinary Portland cement (OPC) used.

**Physical Properties**				
Specific Gravity	Blaine (cm^2^/g)	Setting Time (min)		Loss on Ignition (%)
		Initial	Final	
3.15	4022	136	190	3.3
**Chemical Composition (%)**				
CaO	SiO_2_	Al_2_O_3_	SO_3_	Fe_2_O_3_
70	17	3.9	3.6	3.2

**Table 3:** The specifications of CSWFs used.

Specifications	Chopped Steel Wool Fibers
Length and diameter	4 mm (max) and 25 µm (max)
Specific gravity	7800 kg/m^3^
Tensile strength	966–1242 MPa
Young’s modulus	200–210 GPa

## Data Availability

The data presented in this study are available on request from the corresponding author.
